# 

**Published:** 1915-06-19

**Authors:** 


### Burial Two Years After Death.

An eccentric will, in which the Walthamstow Hospital figured as joint-residuary legatee with the National Lifeboat Institution, is recalled by the recent burial of Mrs. Harriet Hooker, of Highams Park, and the interment was remarkable in that it took place two years after the death. By her will Mrs. Hooker gave detailed instructions for her burial, which was to take place in a mausoleum to be erected in the Walthamstow Cemetery, and during the time the necessary preparations for the interment have been proceeding the body, which was embalmed, has been resting in a mortuary from June 1913 to June 1915.

Miss Gaskell Seed, of Bradford, has been appointed by the Manchester Corporation to the new post of superintendent of women health visitors under the Sanitary Committee. In connection with this appointment it will be recalled that there is a new scheme for the establishment of four baby clinics in Manchester.

The Chelsea Hospital for Women, which is giving special facilities to the near relatives of those at the Front and to Belgian refugees, has received £250 from the trustees of Smith's (Kensington) Charity for its rebuilding fund.

RATES OF SUBSCRIPTION: Twelve months, 6/6; Six months, 4/-; Three months, 2/-, post free to any address in the United Kingdom. Abroad, Twelve months, 8/8; Six months, 5/-; Three months, 2/6, post free.—Address The Subscription Dept., "THE HOSPITAL," The Hospital Building, 28/29 Southampton Street, Strand, London, W.C.

**Twenty-Sixth Year of Publication.** Over 1,000 pages. In Scarlet Cloth. **Price 10/6 net; by post, 10/11**

### BURDETT'S
### HOSPITALS and CHARITIES, 1915.

The Year Book of Philanthropy and Hospital Annual.

**Edited by Sir HENRY BURDETT, K.C.B., K.C.V.O.**

"The Ideal of what a work of reference ought to be."—*The Times.*

**The volume for 1915 is the 26th Issue of "Hospitals and Charities," and contains SPECIALLY WRITTEN CHAPTERS on:—**

The Volume of Charity, 1913. The Future of the Hospitals. The King's Fund and the League of Mercy. The Nursing Department in 1913 and its Cost. Hospital Sunday. Hospital Saturday. Missions. Orphanages. The Blind. The Deaf and Dumb. Hospital Finance—Income, 1913; Expenditure, 1913. The United States, Canada, Australasia, and India. Convalescent Institutions. Hospital Construction, 1914.

**ALL WITH CAREFULLY ANALYSED FIGURES, AND A DIRECTORY OF**

Medical Universities, Colleges, and Schools.—London Hospitals.—London Dispensaries.—Metropolitan Asylums Board Hospitals.—Poor-Law Infirmaries in London and the Provinces.—Provincial Hospitals.—Provincial Infectious Hospitals.—Provincial Dispensaries.—Scotch Hospitals.—Scotch Dispensaries.—Irish Hospitals.—Lunacy Board and Lunatic Asylums: Indian, British, and Colonial.—Military and Naval Hospitals.—Convalescent Homes.—Sanatoria for Consumption.—Retreats for Inebriates.—Private Asylums.—Nursing Institutions.—Indian, Colonial, and American Hospitals.—Hospitals for Insane in the United States of America.—Administrative and Collective Societies.—Missionary and Religious Societies.—Orphanages, Homes, and Charities.—Institutions for the Blind.—Institutions for the Deaf and Dumb.—London County Council Centres for Blind Children.—London and Provincial Institutions for the Deaf and Dumb.—London County Council Centres for Deaf and Dumb Children.—Hospitals and Homes for Incurables.—Establishments for the Relief of Epilepsy.—Vaccine Lymph Establishments.—Reformatory and Industrial Schools.

**Obtainable of all Booksellers.** **Publishers: The Scientific Press, Ltd., London.**

